# Tribbles homolog 2 (Trib2), a pseudo serine/threonine kinase in tumorigenesis and stem cell fate decisions

**DOI:** 10.1186/s12964-021-00725-y

**Published:** 2021-04-01

**Authors:** Yu Fang, Angelina Olegovna Zekiy, Farhoodeh Ghaedrahmati, Anton Timoshin, Maryam Farzaneh, Amir Anbiyaiee, Seyed Esmaeil Khoshnam

**Affiliations:** 1 Anyang Center for Chemical and Pharmaceutical Engineering, College of Chemistry and Chemical Engineering, Anyang Normal University, Anyang, 455000 Henan People’s Republic of China; 2 Key Laboratory of New Opto-Electronic Functional Materials of Henan Province, College of Chemistry and Chemical Engineering, Anyang Normal University, Anyang, 455000 Henan People’s Republic of China; 3 Department of Prosthetic Dentistry, Sechenov First Moscow State Medical University, Moscow, Russia; 4 Department of Immunology, School of Medicine, Isfahan University of Medical Sciences, Isfahan, Iran; 5 Independent researcher, Moscow, Russia; 6 Fertility, Infertility and Perinatology Research Center, Ahvaz Jundishapur University of Medical Sciences, Ahvaz, Iran; 7 Department of Surgery, School of Medicine, Ahvaz Jundishapur University of Medical Sciences, 61357-15794 Ahvaz, Iran; 8 Persian Gulf Physiology Research Center, Medical Basic Sciences Research Institute, Ahvaz Jundishapur University of Medical Sciences, Ahvaz, Iran

**Keywords:** Pluripotent stem cells, Tribbles homolog 2, Tumorigenesis, Pluripotency, Reprogramming, Stem cell fate, Regenerative medicine

## Abstract

The family of Tribbles proteins play many critical nonenzymatic roles and regulate a wide range of key signaling pathways. Tribbles homolog 2 (Trib2) is a pseudo serine/threonine kinase that functions as a scaffold or adaptor in various physiological and pathological processes. Trib2 can interact with E3 ubiquitin ligases and control protein stability of downstream effectors. This protein is induced by mitogens and enhances the propagation of several cancer cells, including myeloid leukemia, liver, lung, skin, bone, brain, and pancreatic. Thus, Trib2 can be a predictive and valuable biomarker for the diagnosis and treatment of cancer. Recent studies have illustrated that Trib2 plays a major role in cell fate determination of stem cells. Stem cells have the capacity to self-renew and differentiate into specific cell types. Stem cells are important sources for cell-based regenerative medicine and drug screening. Trib2 has been found to increase the self-renewal ability of embryonic stem cells, the reprogramming efficiency of somatic cells, and chondrogenesis. In this review, we will focus on the recent advances of Trib2 function in tumorigenesis and stem cell fate decisions.

**Video abstract**

**Supplementary Information:**

The online version contains supplementary material available at 10.1186/s12964-021-00725-y.

## Background

Tribbles homolog 2 (Trib2) is a pseudo serine/threonine kinase and a member of the Tribbles family that functions as a scaffold or adaptor in signaling pathways in a number of physiological and pathological processes [1–3]. The family of Tribbles proteins play many critical nonenzymatic roles and regulate a wide range of key signaling pathways such as mitogen-activated protein kinase (MAPKs), nuclear factor-κB (NF-kB), PI3K/AKT, and activating transcription factor 4 (ATF4) in healthy and pathological processes [4–6]. Trib2 can interact with E3 ubiquitin ligases and control protein stability of downstream effectors [7]. Trib2 as a mitosis blocker regulates various cellular processes, including germ cell development, apoptosis, proliferation, lineage specification, reproduction, inflammation, innate immunity, and drug resistance [8–11]. It is known that Trib2 has diverse roles in neurological disorders, metabolic diseases, autoimmune and inflammatory diseases, arthritis, and a number of cancers (chronic myeloid leukemia, liver, melanoma, and ovarian) [3, 12, 13]. In human cancer, Trib2 as a cancer-associated pseudokinase and novel oncogene can enhance cell proliferation and stimulate cell cycle arrest [14]. Trib2, recently identified as the cause of cancer drug resistance [15, 16]. There is substantial evidence that Trib2 can be a predictive and valuable biomarker for cancer diagnosis and treatment [6, 17]. Recent studies have illustrated that Trib2 plays a major role in cell fate determination of stem cells [2]. Stem cells are undifferentiated cells that have the capacity to self-renew and differentiate into specific cell types [18, 19]. Stem cells are classified into pluripotent (embryonic stem cells (ESCs) and induced pluripotent stem cells (iPSCs)), multipotent (mesenchymal stem cells (MSCs) and hematopoietic stem cells (HSCs)), and unipotent stem cells [20–22]. Human PSCs and MSCs are important sources for cell-based regenerative medicine, tissue engineering, and drug screening [23–25]. Trib2 has been found to increase the self-renewal ability of ESCs, the reprogramming efficiency of somatic cells, and chondrogenesis [2]. In this review, we will focus on the recent advances of Trib2 function in tumorigenesis and stem cell fate decisions.

## Structure and function of Trib2

Tribbles (Trib) is a critical regulator of embryonic development that for the first time identified in Drosophila [26]. In the human genome, the Trib proteins have three homologs, including TRIB1, TRIB2, and TRIB3, with a single kinase-like and highly conserved domain that encodes pseudo‑kinase proteins [9]. Trib2 contains an N-terminal domain (PEST), a C-terminal E3 ligase-binding domain, and a pseudokinase domain with a Ser/Thr protein kinase-like domain (without a canonical ‘DFG’ (metal-binding) motif) [1, 26].

**Fig. 1:**
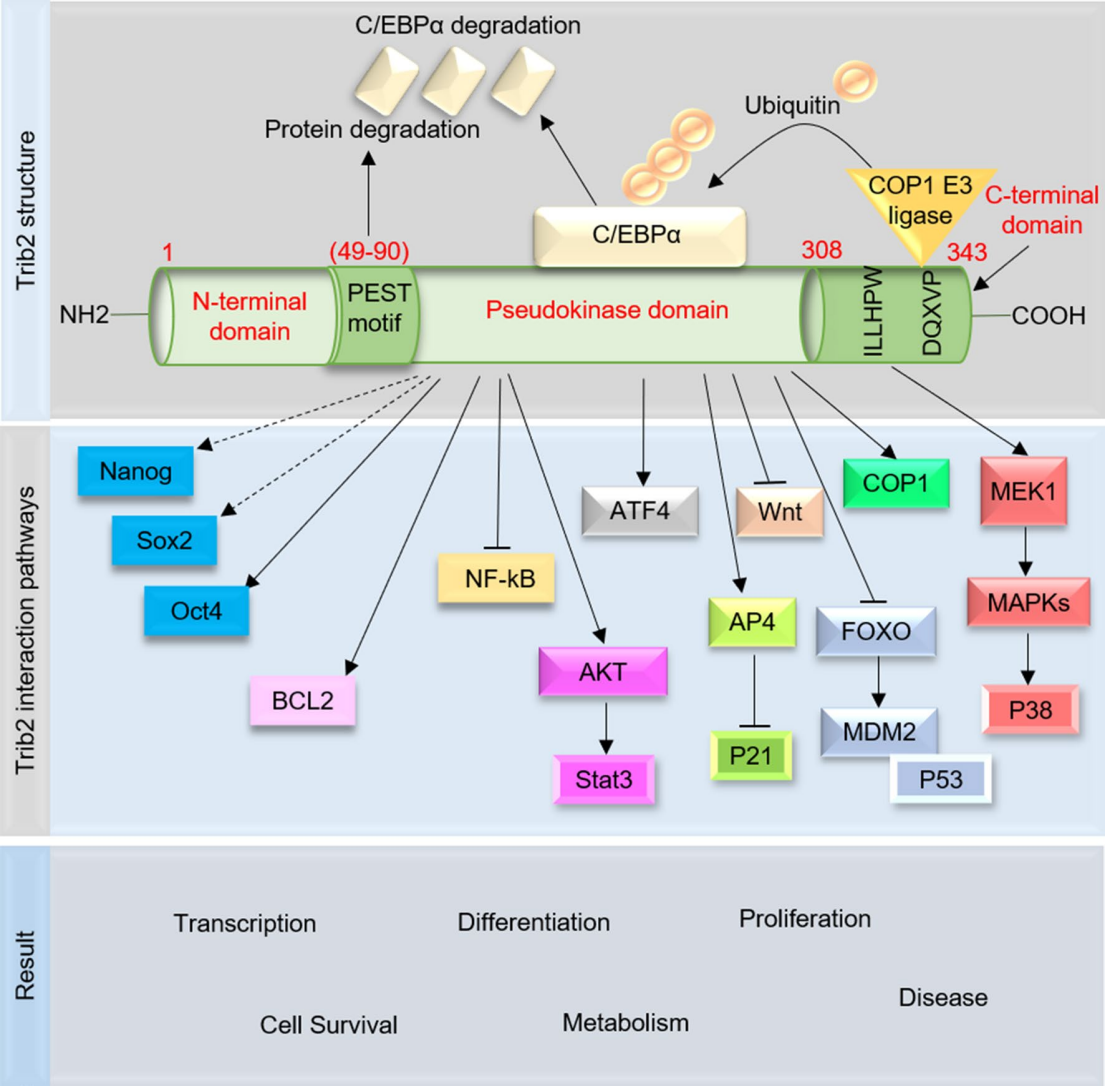
Structural mechanism of Trib2. Trib2 contains an N-terminal domain (PEST), a C-terminal E3 ligase-binding domain, a and pseudokinase domain with a Ser/Thr protein kinase-like domain. The C-terminal domain has the HPW [F/L] motif (targets MAPKK/MEK family members) and the conserved DQXVP [D/E] peptide motif (binds to COP1 E3 ubiquitin ligases). Trib2 can recruit the E3 ligase and induce degradation of CCAAT/enhancer-binding protein β (C/EBPβ). Trib2 regulates a wide range of key signaling pathways such as mitogen-activated protein kinase (MAPKs), nuclear factor-κB (NF-kB), protein kinase B (PKB or AKT), activating transcription factor 4 (ATF4), B-cell lymphoma 2 (BCL2), FOXO (forkhead box protein O), and activating enhancer-binding protein 4 (AP4)

The C-terminal domain has the HPW [F/L] motif (targets MAPKK/MEK family members) and the conserved DQXVP [D/E] peptide motif (binds to COP1 E3 ubiquitin ligases) [27]. The pseudokinase domain has a unique cysteine-rich C-helix that binds with E3 ubiquitin ligases [28] (Fig. 1).

TRIB proteins through E3 ligase-dependent ubiquitination (pseudokinase mechanism) and scaffolding (with the MAPK and AKT pathway) have the potential to control cell proliferation, apoptosis, survival, and differentiation [27]. The family of Tribbles proteins can regulate a wide range of signaling pathways such as mitogen-activated protein kinase (MAPKs), nuclear factor-κB (NF-kB), protein kinase B (PKB or AKT), and activating transcription factor 4 (ATF4) [4–6]. In the Toll-like receptor (TLR) signaling pathway, Trib1 negatively controls the expression of CCAAT/enhancer-binding protein β (C/EBPβ) [29]. Trib1 and Trib2 are able to recruit the E3 ligase and induce C/EBPα and C/EBPβ degradation [30, 31]. Therefore, TRIBs through the degradation of C/EBP transcription factors promote oncogenesis [6]. Trib3 has been shown to regulate serine-threonine kinase AKT (PKB), p65/rel A, and activating ATF4 [32–34]. Trib1 and Trib3 in response to various pro-inflammatory stimuli trigger the MAP kinase pathway and regulate the activator protein 1 (AP-1)-related pathway [35].

Little information is known about the role of Trib2 in mammals [9, 27]. Trib2 has a low affinity for ATP and further structural studies are required for the ATP-binding status of this protein [6, 14]. Trib2 via a ubiquitin- and proteasome-dependent pathway regulates the cell cycle in human cells [31, 36]. It should be noted that both the Trib2 kinase domain and COP1 binding are essential for the ability of Trib2 to degrade C/EBPα [37]. Trib2 has been reported to be a pro-apoptotic molecule that stimulates apoptosis via a caspase-dependent mechanism [38]. Trib2 with the expression of pro- and anti-inflammatory immune modulators appears to be essential for innate immunity [39]. Trib2 as an important regulator of normal hematopoiesis can regulate the differentiation potential of intrathymic precursors and the checkpoints of thymopoiesis [11]. The function of monocytes and macrophages can be modulated by Trib2 [40, 41]. Trib2 has been shown to regulate the development of T-cell and erythroid cells [42, 43]. This protein as an anti-inflammatory factor negatively regulates NF-κB (p100) activity in the TLR5 pathway [9, 44]. During follicular development, Trib2 increases the activity of the MAPK/ERK pathway and regulates granulosa cells (GCs) proliferation and function [45].

## Different roles of Trib2 in tumorigenesis

Trib2 is a cancer-associated pseudokinase that can be induced by mitogens and enhances the propagation of several cancer cells, including myeloid leukemia, liver, lung, skin, bone, brain, and pancreatic [10, 46] (Table 1).

**Table 1:** Pro-tumor and anti-tumor roles of TRIB2

Disease	Mechanism of action	Result	References
Pro-tumor			
AML	Suppresses C/EBPα expression	Enhances AML progression	[50]
	Enhances the expression of anti-apoptotic BCL2	Enhances AML progression	[51]
CML	Stimulates the ERK pathway	Increases cell proliferation and drug resistance	[12]
T-ALL	Decreases C/EBPα expression	Promotes the growth and maintenance of T-ALL cells	[55]
Liver cancer	Increases YAP stabilization	Promotes cancer cell proliferation	[56]
	Interacts with PCBP2 and triggers the UPS	Reduces Ub flux and decrease the oxidative damage	[58]
Lung cancer	Decreases expression levels of C/EBPα	Accelerates cell proliferation and tumor growth	[59]
Malignant melanomas	Suppresses FOXO	Promotes cell proliferation, colony formation, maintenance, and progression	[63]
Human melanoma tissues and cell line		Migration and invasion	[64]
OS cell line		Enhances the malignant capacity	[10]
CRC	Suppresses p21 expression	Improves cell growth and progression, and block cellular senescence	[14]
GBM	Interacts with MAP3K1	Enhances resistance to chemotherapy and radiotherapy	[8]
Pancreatic cancer tissue	Suppresses the p53/MDM2 complex	Promotes resistance to anti-cancer therapy	[66]
LSCC	Interacts with XIST	Enhances proliferation and migration	[69]
OSCC	Interacts with TRIM	Facilitates the development of OSCC	[71]
Anti-tumor			
Myeloid leukemia	Suppresses the Wnt pathway, stimulates activation of p38 stress signaling	Reduces cell propagation	[53]

*AML* acute myeloid leukemia, *BCL2* B-cell lymphoma 2, *CML* chronic myelogenous leukemia, *T-ALL* T cell acute lymphoblastic leukemia, *YAP* yes-associated protein, *FOXO* Forkhead box protein O, *OS* osteosarcoma, *CRC* colorectal cancer, *GBM* glioblastoma; MAP3K1, *MAP* kinase kinase kinase 1, *LSCC* squamous cell carcinoma cells, *XIST* X inactivate-specific transcript, *OSCC* oral squamous cell carcinoma, *TRIM* tripartite motif, *PCBP2* Poly (rC) binding protein 2, *UPS* Ubiquitin (Ub) proteasome system

In normal murine hematopoiesis, TRIB2 function is necessary for the thymopoietic reaction to oncogenic stress [47]. In hematological malignancies, TRIB2 as a target gene of MEIS1, E2F1, and NOTCH1 participates in acute myeloid leukemia (AML) and T cell acute lymphoblastic leukemia (T-ALL) [42, 48, 49]. In human AML, Trib2 has been reported to be a negative regulator of C/EBPα expression and enhances AML progression [50]. In patient-derived human AML cells, Trib2 enhances the expression of anti-apoptotic B-cell lymphoma 2 (BCL2) [51]. In chronic myelogenous leukemia (CML), Trib2 through the ERK pathway increases cell proliferation and drug resistance [12]. Smad ubiquitination regulatory factor 1 (Smurf1) is a HECT-type E3 ubiquitin ligase that acts as a tumor enhancer or suppressor in various biological processes [52]. Trib2 by regulating the degradation of E3 ubiquitin ligase βTrCP, COP1, and Smurf1 can inhibit the Wnt pathway and reduce cell propagation in myeloid leukemia [53]. In response to stress, TRIB2 as a tumor suppressor stimulates activation of p38 stress signaling in myeloid leukemia [54]. Thus, Trib2 may decrease the ability of leukaemia cells to propagate [54]. In T-ALL, Trib2 as a direct target of Notch1 decreases C/EBPα expression and promotes the growth and maintenance of T-ALL cells [55]. Afatinib is a small-molecule protein kinase inhibitor that can promote the degradation of Trib2 in human AML cells [28].

Trib2 can be a target for the Wnt/β-catenin pathway downstream and regulates liver cancer cell growth and transformation [7]. In liver cancer, Trib2 shows high stability and interacts with βTrCP to accelerate Yes-associated protein (YAP) stabilization (Hippo pathway) and promote cancer cell proliferation [56]. Trib2 with its associated E3 ligases can reduce ubiquitination of transcription factor 4 (TCF4) and β-catenin and decrease Wnt activity [57]. TRIB2 has been identified that interacts with poly (rC) binding protein 2 (PCBP2) and triggers the Ubiquitin (Ub) proteasome system (UPS) to reduce Ub flux and decrease the oxidative damage. Therefore, UPS by increasing oxidative damage might be a suitable target against liver cancer [58].

In lung cancer, Trib2 binds with TRIM21 E3 ligase and decreases expression levels of C/EBPα, which accelerates cell proliferation and tumor growth [59]. Recent literature has reported that miR-511 and miR-1297 as tumor suppressor genes decrease Trib2 expression and reduce lung adenocarcinoma cell proliferation [60]. Substantial evidence has shown that miR-206 and miR-140 are Smad3-related miRNAs that inhibit Trib2 expression, induce cell death, and decrease cell proliferation [61].

In malignant melanomas, Trib2 is overexpressed and inhibited FOXO (forkhead box protein O) tumor suppressor activity [62]. Thus, Trib2 is important in cell proliferation, colony formation, maintenance, and progression of melanoma cells [63]. Recent data revealed that circular RNAs (circRNA)-0084043 can interact with miR-429 and positively regulate TRIB2 expression. Trib2, circRNA-0084043, and miR-429 are the leading causes of migration and invasion in human melanoma tissues and cell lines [64]. Members from the thiazolidinediones (TZDs) family was reported to overcome cell drug resistance in Trib2-positive cancer cells [15]

Trib2 has been found to enhance the malignant capacity of osteosarcoma (OS) cell line (malignant bone tumors). miR-509-5p as a tumor suppressor can target Trib2 and suppress cell propagation and migration in OS cell lines [10].

The p53/p21 pathway is thought to be a critical regulator of the cell cycle and cellular senescence [65]. In colorectal cancer (CRC), Trib2 binds with activating enhancer-binding protein 4 (AP4) to suppress p21 expression, improve cell growth and progression, and block cellular senescence [14].

In Glioblastoma (GBM), Trib2 interacts with MAP kinase kinase kinase 1 (MAP3K1) and enhances resistance to temozolomide (TMZ) chemotherapy and radiotherapy [8].

In primary pancreatic cancer tissue, Trib2 protein has been shown to block FOXO activation, disrupt the p53/MDM2 complex (a negative feedback loop for cancer therapy), stimulate the serine/threonine protein kinase AKT, reduce cell death induced by PI3K inhibitors, and promote resistance to anti-cancer therapy [66, 67]. Recent evidence suggests that ZEB1-AS1 as a long non-coding RNA (lncRNA) by regulating miR-505-3p/TRIB2 axis enhances the growth, viability, and invasion of pancreatic cancer cells [68].

X inactivate-specific transcript (XIST) is a lncRNA, which was recently proposed to interact with miR-125b-5p, promote Trib2 expression, and enhance proliferation and migration of laryngeal squamous cell carcinoma (LSCC) cells [69].

Tripartite motif (TRIM) protein has an important role in the pathogenesis of oral squamous cell carcinoma (OSCC) [70]. TRIM via modulating the TRIB2-MAPK signal axis can promote abnormal expression of interleukin-6 (IL-6) and disrupt TH1/TH2 balance (interferon-gamma (IFN-γ) and IL-4) in T cells [71].

Therefore, Trib2 may be a suitable biomarker for the cancer diagnosis, because it shows high expression in malignant cells [27].

## The function of Trib2 in stem cell fate decisions

Human ESCs are derived from donated pre-implantation embryos and the inner cell mass (ICM) of the blastocyst [72, 73]. A recent study has reported that Trib2 may be necessary for colony formation, alkaline phosphatase (AP) activity, and self-renewal ability of ESCs [2]. Trib2 interacts with Oct4 and regulates the expression of the pluripotency marker genes. Thus, loss of Trib2 expression is associated with differentiation of ESCs [2]. Human iPSCs are ESC-equivalent cells that can be derived by introducing core reprogramming factors (Oct4, Sox2, Nanog, and Klf4 or OSKM) into embryonic fibroblasts [74, 75]. Trib2 plays an important role in the reprogramming of somatic cells [2]. It has been shown that Trib2 knockdown reduces the reprogramming efficiency and the expression of OSKM in the generated cells. While colony formation and AP activity in OSKM/Trib2 transduced cells were higher than cells transduced with the OSKM factors. Trib2 through the Trib2-Oct4 complex can facilitate the generation of iPSCs from somatic cells [2].

**Fig. 2:**
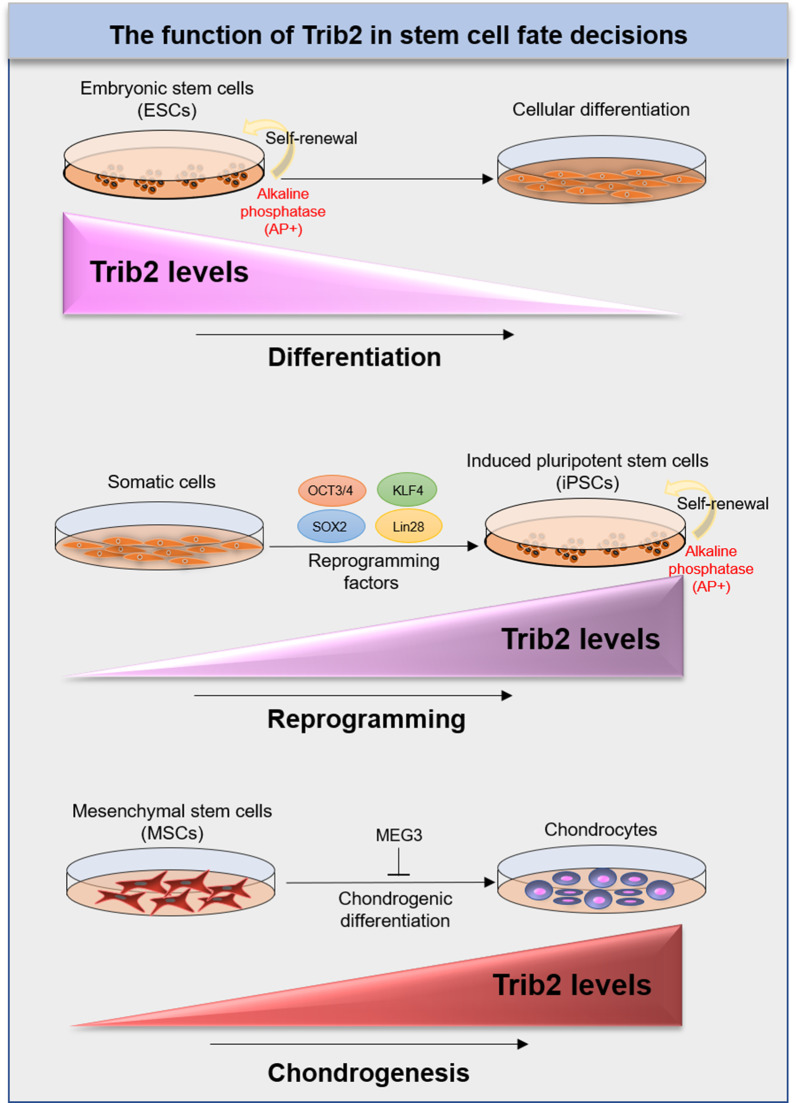
The function of Trib2 in stem cell fate decisions. Human ESCs are derived from donated pre-implantation embryos and the inner cell mass (ICM) of the blastocyst. Human iPSCs are ESC-equivalent cells that can be derived by introducing core reprogramming factors (Oct4, Sox2, Nanog, and Klf4 or OSKM) into embryonic fibroblasts. Trib2 is necessary for colony formation, alkaline phosphatase (AP) activity, and self-renewal ability of ESCs and iPSCs. MEG3 can downregulate Trib2 expression and suppress the chondrogenic differentiation of synovium-derived MSCs

It has been reported that Trib2 through a proteasome-dependent mechanism induces the degradation of C/EBPβ and suppresses adipocyte differentiation at an early stage [76, 77]. The differentiation of myeloid progenitor cells can be suppressed with the MLL-TET1 (MT1) fusion protein. This protein induces Trib2 mRNA and protein expression and decreases C/EBPα expression. Thus, Trib2 is important to keep leukemic cells in an undifferentiated state [78]. Trib2 has been found to increase chondrogenesis from MSCs. MEG3 as a lncRNA has been reported to upregulate enhancer zeste homolog 2 (EZH2) methyltransferase and downregulate Trib2 expression to suppress the chondrogenic differentiation of synovium-derived MSCs [79] (Fig. 2).

## Challenges and prospective

The expression of TRIB2 in tumor tissues and cell lines is significantly increased [66]. High TRIB2 expression was shown to be essential in melanoma progression, lung tumorigenesis, liver and colon tumors [3, 26, 58, 80]. Hence, TRIB2 can be a novel targeted therapeutic and strong candidate against chemoresistant cancers [81]. However, the exact mechanism of TRIB2 as an adaptor protein in cancer is still controversial and remains unclear [82]. Therefore, multiple genetic and epigenetic mutations should be assessed to identify the specific interaction of TRIB2 with partner proteins [83].

## Conclusion

We have highlighted various studies, which provide evidence of Trib2 protein as an attractive target for cancer therapy. Although Trib2 has a key role in multiple physiological and pathological processes, much effort will be required to find its relevance to stem cell fate decisions. It can be concluded that Trib2 may represent a potential target in basic research and cancer treatment.

## Data Availability

The datasets used and/or analyzed during the current study are available from the corresponding author on reasonable request.

## Abbreviations

AP: Alkaline phosphatase
AP-1: Activator protein 1
AML: Acute myeloid leukemia
ATF4: Activating transcription factor 4
BCL2: B-cell lymphoma 2
C/EBPβ: CCAAT/enhancer-binding protein β
CML: Chronic myelogenous leukemia
ESCs: Embryonic stem cells
EZH2: Enhancer zeste homolog 2
FOXO: Forkhead box protein O
GCs: Granulosa cells
HSCs: Hematopoietic stem cells
IPSCs: Induced pluripotent stem cells
MAPKs: Mitogen-activated protein kinase
MT1: MLL-TET1
MSCs: Mesenchymal stem cells
NF-kB: Nuclear factor-κB
Smurf1: Smad ubiquitination regulatory factor 1
TCF4: Transcription factor 4
TLR: Toll-like receptor
T-ALL: T cell acute lymphoblastic leukemia
Trib2: Tribbles homolog 2
TZDs: Thiazolidinediones
XIST: X inactivate-specific transcript
YAP: Yes-associated protein
